# Trend towards varying combinatorial centromere association in morphologically identical clusters in Purkinje neurons

**DOI:** 10.1186/1475-9268-5-1

**Published:** 2006-12-07

**Authors:** Kunjumon I Vadakkan, Baoxiang Li, Umberto De Boni

**Affiliations:** 1 Department of Physiology and Collaborative Program in Neuroscience, Faculty of Medicine, University of Toronto, Toronto, ON, M5S1A8, Canada

## Abstract

Neurons with similar morphology and neurotransmitter content located at a specific brain region may be part of the same or functionally separate networks. To address the question whether morphologically similar neurons have similar structural architecture at the chromosomal level, we studied Purkinje neurons in the cerebellum. Previous studies have shown that in Purkinje neurons centromeres of several chromosomes form clusters and that the number and size of these clusters remain stable in the adult brain. We examined whether the same set of centromeres form clusters in all the Purkinje neurons. Fluorescent *in situ *hybridization (FISH) with chromosome-specific para-centromeric probes provided an indirect evidence for a trend towards varying contributions from different chromosomes forming the centromeric clusters in adjacent Purkinje neurons. The results of the study indicate that the individual Purkinje neurons are likely unique in inter-chromosomal spatial associations.

## Background

Different plasticity changes in neurons may lead to expression from gene loci belonging to different chromosomes. For example, subunit genes for NMDA receptor reside in different chromosomes (in mouse, NR1 and NR2B in chromosome 2; NR2A in chromosome 16). However, the process of optimization of an organized synthetic process in controlling a proportional transcriptional control from different chromosomes in neurons is not yet known. With the exception of neurogenesis, majority of the neurons in adult nervous system have completed cell division, fully differentiated and permanently arrested in interphase. Do neurons of similar morphological structure and neurotransmitter contents have similar structural architecture at the chromosomal level? Recent reports strongly suggest associations between gene loci present in different chromosomes [1-5]. The possible implications of this process in neuronal nuclei may have important functional significance. To address these questions, previous studies were carried out in Purkinje neurons in cerebellum [6-8]. These studies have shown that adjacent Purkinje neurons have similar spatial distribution of centromeric and telomeric clusters. However, similarities at the sub-chromosomal level among morphologically identical neurons were not examined. In pathological conditions, alteration in sub-chromosomal organization is reported. For example, the spatial distribution of satellite DNA is altered in neurons in epileptogenic foci [9] and in hippocampal neurons during experimental long term potentiation (LTP) paradigm [10]. Since mouse chromosomes except Y are telocentric, clustering of the centromeres is a suitable model to study similarities and variations at the sub-chromosomal level among morphologically similar neurons.

In Purkinje neurons of the adult mouse, the number of centromeric kinetochore clusters and spatial distribution, as detected by immunocytochemistry [8] were shown to be routinely similar leading to the possibility that the same chromosomes contribute their centromeres to a given cluster. Accordingly, we hypothesized that a pair of centromeres has two possibilities in their spatial positions. One, they may always form part of a cluster and therefore are always associated. Second, if they are part of two separate clusters, they never cluster. Our recent work has shown routine clustering of centromeres of one homologue each of chromosomes 2 and 11 in Purkinje neurons [11]. To test whether centromere clustering between other chromosomes in Purkinje neurons has chromosome-chromosome specificity, we carried out FISH using chromosome-specific para-centromeric sequences from randomly picked pairs of chromosomes.

## Results

**Figure 1:**
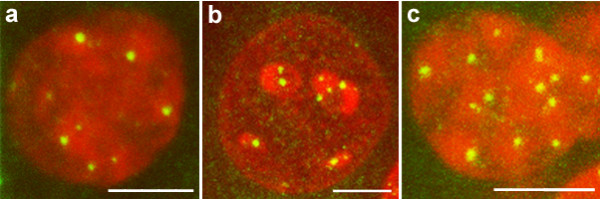
Superimposed stacks of confocal sections showing centromeric clusters (green signals) in representative nuclei (red) of a) cerebral cortical pyramidal neuron b) cerebellar Purkinje neuron and c) cerebellar granule neuron of mouse observed by immunocytochemical staining of the kinetochore proteins. DNA in the nuclei is stained with ethidium bromide (red). Scale bar in all the figures = 5 μm

**Figure 2:**
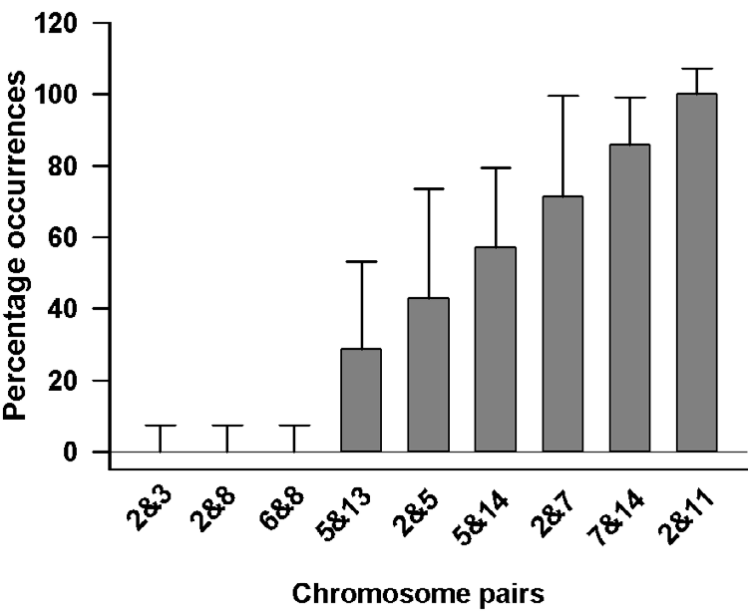
Frequency histogram showing centromere clustering of specific chromosome pairs in the Purkinje neurons (chromosome pairs 2&3, 2&8, 6&8 (n = 7 cells); 2&5, 2&7 (n = 10); 5&13 (n = 13); 5&14 (n = 19); 7&14 (n = 26); 2&11 (n = 29). Wide range of clustering is observed between one pair of homologues indicating trend towards varying combinatorial centromere association. Note that the second pair of homologues did not show clustering in any of the chromosome pairs studied. 95% confidence intervals are shown as error bars.

Using immunocytochemistry to the kinetochrore proteins associated with centromeres, our results show that the number of centromere clusters in the nuclei of cortical pyramidal, cerebellar Purkinje and cerebellar granule neurons (Fig. 1) are much less than the chromosome complement for the species (40 in number). By comparing with the fibroblast cell nucleus, we have previously reported that the increased signal size of the centromeres observed in Purkinje neuronal nucleus results from clustering of multiple centromeres [8]. Using FISH experiments, simultaneous application of probes for two randomly selected centromeres resulted in four discrete signals in Purkinje neurons for all the nine pairs of chromosomes tested. Measurements of inter-signal distances showed that one homologue each of the chromosome pairs exhibited wide range of clustering (Fig. 2). We have previously reported the routine clustering of chromosome pairs 2 and 11 in Purkinje neurons [11]. In contrast to this, pairs 2&3, 2&8 and 6&8 routinely did not show clustering of centromeres. Whenever clustering was observed, it was restricted to one pair of homologous chromosomes; the second pair did not cluster in any of the cells examined. In summary, the extent of centromere clustering among pairs of chromosomes studied in Purkinje neurons is limited to only one pair of homologues and the percentage occurrence showed a wide range of variation between zero and hundred suggesting varying combinatorial association.

## Discussion

Functional regulation in cells involves different structural changes in chromosomes, DNA and proteins. In neurons, plasticity as well as metabolic requirements are also controlled by mechanisms ranging from regulation of subunit assembly of multi-unit proteins [12] to generation of hybrid metabolites from limited set of genes [13]. Controls on gene expression by centromeric heterochromatin-mediated silencing was shown to relocate a gene into its proximity [14]. Centromeric repeat homology is also found in small RNAs in RNA interference (RNAi) effecter complex RITZ [15]. In these contexts, the clustered centromeric heterochromatin in Purkinje neurons [8,11,16] may have important functional roles. The size and number of centromeric clusters in adult Purkinje neurons remained almost the same with less variability [8] leading to the hypothesis that it is the same chromosomes that always contribute their centromeric domains to a given cluster. We haven't directly assessed the composition of a particular cluster. Instead our approach was an indirect one by using pairs of centromere probes to detect the spatial relationship. According to our hypothesis a pair of centromeres will either always cluster or not cluster at all. Contrary to our expectations, the results have shown a trend towards wide range of clustering. The reasons for this remain unknown. Epigenetic variations in expression profiles of genes may account for the variability observed since heterochromatin may play a major role in epigenetics [17]. Some centromeres show higher levels of clustering indicating the possibility that clustering of at least some chromosomes may be obligatory for maintaining and/or regulating housekeeping gene expression; while others are facultative depending on the varying functional demands made on these neurons or depending on the specific network connections. In addition, the structural variations may contribute to the functional demands on morphologically similar neurons representing different somatic locations of the cerebellar homunculus [18,19]. Since the present work was done using parasagital sections from the cerebellum, the results may be interpreted as that of a parasagital topographical feature, until further investigations in other planes are carried out.

Previous work has shown that centromeres of chromosomes 2 and 11 were detected in close proximity in Purkinje neurons [11]. The results of the present work show that the additional chromosomes that contribute their centromeres to the same cluster that contains centromeres of chromosomes 2 and 11 vary. For example, the centromere of chromosome 2 has varying clustering with centromeres of chromosomes 3 and 8. It is still not known whether centromeres of some chromosomes are interchangeable with respect to the centromere cluster formation. Though the present work neither tested all the centromeric clusters nor analyzed one specific cluster to test the hypothesis that these are the same centromeres that contribute to a given cluster, our results using a pair of centromeric probes provides an indirect evidence that there is a varying contribution from different chromosomes towards a given centromeric cluster. Future experiments using primers to pericentromeric regions by new methods like capturing chromosome conformation (3C) [2,20] will help to explore the structural details.

## Conclusion

The present study examined whether the centromere clusters in morphologically similar Purkinje neurons consist of same set of centromeres. Fluorescent *in situ *hybridization (FISH) with chromosome-specific para-centromeric probes provided an indirect evidence for a trend towards varying contributions from different chromosomes forming the centromeric clusters in Purkinje neurons. This structural organization among morphologically identical neurons may have physiologically important roles.

## Methods

Experiments were performed under protocols approved by the University of Toronto animal care committee. Briefly, formaldehyde fixed (30 min) cerebellum of CD1 mice were Vibratome sectioned at 50 μm in a parasagital plane and used for both immunocytochemistry and FISH. For immunocytochemistry, all steps were carried out on floating sections. Sections were incubated in RNAase A (100 μg/mL PBS, 37°C, 2 hr), washed (PBS, 10 min) and blocked (4% BSA, PBS, 0.02% sodium azide, 2 hr, RT). The sections were then incubated in human CREST-type scleroderma anti-centromeric autoimmune serum (a gift from Dr. L. Rubin, 1:1000 in PBS, 2% Triton-X 100, 0.02% sodium azide, 3.2% BSA, 37°C, 24 hr, cross referenced with Centre for Disease Control Reference Serum, # 8). Following incubation, sections were washed (PBS, 3 × 20 min) and blocked again (4% BSA, 2 hr, RT) and stained by Alexa-conjugated goat anti-human IgG (Molecular Probes, 1:200, PBS, 0.02% sodium azide, 24 hr, 37°C). Sections were washed, (PBS, 3 × 10 min), counterstained (ethidium bromide 1 μg/mL, PBS, 10 min) and mounted.

For FISH experiments cerebellar vermis of adult CD1 mice was fixed (90 min) in 4% paraformaldehyde (PBS, pH 7.3). All the steps of the FISH experiments were carried out on sections that were adhered to aminopropyltriethoxysilane-coated glass cover slips. Sections were deproteinized (0.2 N HCl, 60 min, RT), washed (PBS, 3 × 5 min), permeabilized (1% Triton X-100, 1% Igepal CA-630, PBS, overnight, RT) and washed again (PBS, 3 × 5 min). Following incubation with RNAse-A (100 μg/mL, PBS, 37°C, 2 hrs), the sections were washed (PBS, 3 × 5 min), and equilibrated with Proteinase K buffer (1 mM Tris-HCl (pH 7.8), 0.5 mM EDTA, 0.05% SDS, 5 min, RT) followed by digestion with Proteinase K (Roche # 1964372), 40 μg/mL, Proteinase K buffer, 35 min, RT). Washed (PBS, 3 × 5 min) and digestion products were removed (1% Triton X-100, 1% Igepal CA-630, PBS, 20 min) and washed (PBS, 3 × 5 min). After equilibration (70% formamide, 2× SSC, overnight, RT), DNA in the cells was denatured (pre-heated 70% formamide, 2× SSC, 70°C, 12.5 min) and quickly chilled (ice-cold 50% formamide, 2× SSC, 10 min). The para-centromeric, chromosome-specific probes [21] were obtained as BAC clones (Research Genetics, MB 11300; now available from Open Biosystems, BMM 1036). The identities of the BAC clones were confirmed by *in situ *hybridization. BAC plasmids were isolated and haptens (biotinylated dATP (Invitrogen # 19524-016) or dinitrophenol (DNP)-modified dUTP (PerkinElmer, NEL 551) were incorporated using standard nick translation protocols. The labeled probe (200 nanograms; size 200 base pairs or less to facilitate access to the nuclear interior) was ethanol precipitated with herring sperm DNA and mouse cot-1 DNA in a ratio of 1:50:10 and then air-dried. The DNA pellet was dissolved in 5 μL, 100% formamide and an equal volume of 4× SSC, 20% dextran sulphate was added. Before use, the probe was denatured (boiling water bath, 2 min) and immediately transferred to a water bath (37°C, 30 min) for pre-annealing of cot-1 DNA with repetitive sequences in genomic DNA, to facilitate subsequent hybridization to specific sequences.

For hybridization, 10 μL of the probe, still at 37°C, were placed on a depression-glass slide (well volume 8 μL, EMS Sciences, PA). The cover slip with the denatured section attached was then inverted onto the well, sealed with rubber cement and allowed to hybridize in a humidified chamber (37°C, overnight). After hybridization, the cover slip was placed in pre-heated (45°C) post-hybridization washing solution (3 × 10 min, 50% formamide, 2× SSC) and washed (SSC, 45°C, 3 × 10 min). Sections were blocked (4% BSA, PBS, 2 hr, RT) and incubated either in anti-biotin monoclonal antibody (Roche # 1297597, 1:1000, 4% BSA, PBS, 0.02% sodium azide) or goat anti-DNP antibody (Bethyl Lab, A150-117A-1, 1:1000, 4% BSA, PBS, 0.02% sodium azide, overnight, 37°C). Sections were washed (PBS, 3 × 5 min), blocked and labeled. For multi-color FISH, Alexa Fluor^® ^488 (Molecular Probes, #A-21202, donkey-anti-mouse, 1:200, 4% BSA, PBS, 0.02% sodium azide) was used as the secondary antibody for biotin, whereas DNP was detected by Alexa Fluor^® ^647, donkey anti-goat IgG (Molecular Probes A-21447; 1:200, 4% BSA, PBS, 0.02% sodium azide, 2 hr). Sections were then washed (PBS, 3 × 5 min), nuclei counterstained (ethidium bromide, 1 μg/mL, PBS, 10 min) and mounted. Carl Zeiss 510 confocal microscope multi-track facility was used to collect signals of different colors. Nuclei were optically sectioned at 0.33 μm focal steps, using 100×, 1.3 numeric aperture oil-immersion objective. Image galleries were used to generate 3D volume of the nuclei and the relative spatial positions of the signals were measured. The sizes of centromere clusters were measured and their diameters were used as the maximum possible distance at which the centromeres can be present, but still be part of a centromere cluster [11]. Signals that were present at the central chromocenter of Purkinje neuron nuclei were categorized as part of a centromere cluster if the distance between signals was ≤ 3.5 μm and those outside the central chromocenter were categorized as part of a centromere cluster if the distance between the signals was ≤ 1.54 μm. 95% confidence intervals for the observed percentage clustering were calculated, since we used random samples of the Purkinje neuron population, and are plotted as error bars.

## Competing interests

The author(s) declare that they have no competing interests.

## Authors' contributions

UDB and KIV conceived and designed the experiments. KIV and BL performed the experiments. UDB and KIV collected the data and wrote the manuscript.
